# Emotional State and Pain Experience During Orthodontic Appliance Removal: Evaluation of Four Debonding Protocols

**DOI:** 10.3390/dj14060386

**Published:** 2026-06-22

**Authors:** Elsa Conde-Disla, María José González-Olmo, Marta Olmos-Valverde, Ana Ruiz-Guillén, Martín Romero Maroto

**Affiliations:** 1 Department of Orthodontics, Rey Juan Carlos University, 28922 Madrid, Spain; e.conded@alumnos.urjc.es (E.C.-D.); marta.olmos.valverde@urjc.es (M.O.-V.); martin.romero@urjc.es (M.R.M.); 2 Department of Pediatric Dentistry, Rey Juan Carlos University, 28922 Madrid, Spain; ana.guillen@urjc.es

**Keywords:** orthodontic debonding, pain perception, interocclusal stabilization, negative affect, anxiety, biopsychosocial model

## Abstract

**Background:** Pain during orthodontic debonding is a common clinical concern. Although previous studies have mainly focused on mechanical approaches to reduce discomfort, the influence of emotional characteristics of patients on pain perception remains insufficiently explored. In this study, we aimed to evaluate the association between pain perception, emotional affect, and anxiety during orthodontic bracket removal using different clinical protocols. **Methods:** A prospective observational comparative study was conducted at Rey Juan Carlos University (Madrid, Spain). A total of 140 orthodontic patients underwent bracket removal according to four routine clinical protocols determined by clinical scheduling: ligated with interocclusal cotton rolls (used for tooth stabilization), non-ligated with cotton rolls, ligated without cotton rolls, and non-ligated without cotton rolls. Pain intensity was assessed using a Visual Analog Scale (VAS) immediately before (T0) and after (T1) bracket removal. Baseline pain (T0) was recorded to control for pre-existing discomfort. Anxiety and emotional affect were measured using the State–Trait Anxiety Inventory (STAI) and the Positive and Negative Affect Schedule (PANAS), respectively. Statistical analyses included ANOVA, factorial ANCOVA adjusted for baseline pain, and multivariable regression models. **Results:** No significant baseline differences were observed among groups. The highest post-debonding pain scores were found in the group without cotton rolls and without ligatures. ANCOVA revealed a significant main effect of cotton roll use, with lower adjusted pain scores in patients treated with cotton rolls, whereas ligation showed no statistically significant independent effect. In multivariable regression analyses, baseline pain, age, and negative affect were independently associated with higher post-debonding pain. **Conclusions:** Within the limitations of a non-randomized design, cotton roll use was associated with lower post-debonding pain, whereas ligation appeared to have a limited influence. Patient-related factors—particularly negative affect, age, and baseline pain—were also associated with pain perception, supporting a biopsychosocial perspective. These findings should be interpreted as exploratory evidence rather than causal effects.

## 1. Introduction

Orthodontic pain is a frequent clinical symptom resulting from the acute inflammatory response triggered by vascular occlusion and tissue remodeling following the application of orthodontic forces [1]. Patients typically experience pain at three key stages of treatment: appliance placement, periodic activation, and bracket removal. Notably, pain following appliance placement is primarily related to the biological processes underlying tooth movement [2,3,4], and the intensity of this pain may be influenced by multiple factors, including age, sex, psychological status, clinical procedures, and the use of interocclusal materials [5].

During debonding, mechanical factors may play a relevant role in pain perception. Stabilizing teeth by asking patients to bite on an interocclusal material has been proposed as a method to reduce discomfort by improving force distribution [6,7]. Another approach that could contribute to pain reduction is tooth ligation during bracket removal. The four debonding protocols evaluated in this study were selected based on these clinically relevant variables, namely the use of interocclusal materials and ligation, allowing assessment of their individual and combined effects on pain perception.

Pain perception is also modulated by psychological factors. Negative emotions, anxiety, and prior experiences can amplify pain, whereas positive affect may attenuate it [8,9]. In addition, the debonding appointment represents a meaningful transitional moment for many patients, which may evoke heightened emotional responses. Anticipatory anxiety related to uncertainty, fear of pain, or concerns about dental appearance after appliance removal may increase negative emotional states and, consequently, pain perception, as has been observed in patients undergoing medical procedures such as blood tests or diagnostic examinations [10].

Moreover, changes in self-perception following bracket removal—such as increased attention to dental sensitivity, enamel irregularities, or unmet esthetic expectations—may further contribute to emotional distress [11].

These cognitive and emotional factors could amplify nociceptive processing, reinforcing the link between anxiety, negative affect, and pain during debonding procedures. Although the neurobiological mechanisms linking emotion and pain are not fully understood, their interaction highlights the importance of a biopsychosocial approach to orthodontic pain [12]. Despite existing evidence on pain during orthodontic treatment, limited research has examined the combined effect of mechanical and emotional factors during bracket removal [2,13].

The present study aimed to compare pain levels before and after bracket debonding across different protocols. Additionally, it sought to evaluate the independent and combined effects of cotton roll use and ligation status on post-debonding pain, adjusting for baseline pain. Finally, the study investigated the predictive role of clinical (baseline pain), demographic (age), and psychological (negative affect) variables in post-debonding pain perception.

## 2. Materials and Methods

### 2.1. Study Design and Setting

A prospective observational comparative study was conducted at the Rey Juan Carlos University clinic between September 2022 and July 2024. The sample size was calculated to ensure a statistical power of 80% and a significance level of 0.05, allowing significant differences in pain perception to be detected during bracket removal among the intervention groups. Considering a comparative design with four parallel groups and assuming a medium effect size (f = 0.25) based on previous studies [14,15], a minimum required sample of 128 participants was estimated using G*Power software 3.1 (Heinrich Heine University, Dusseldorf, Germany). To guarantee achievement of the calculated sample size, a larger number of patients was initially assessed. Of all subjects who completed their orthodontic treatment over a 24-month period, 159 patients were screened for eligibility. Of these, 140 met the inclusion criteria and agreed to participate in the study, while the remaining individuals were excluded for not meeting the established criteria or declining participation. This strategy ensured the required sample size, anticipating potential losses or exclusions prior to definitive inclusion. All participants were informed about the objectives and nature of the study and signed written informed consent before enrollment.

Specific inclusion and exclusion criteria were established for participant selection. Eligible candidates were required to be between 12 and 79 years of age, to possess sufficient maturity to understand and complete the required questionnaires, and to not have taken analgesic or anti-inflammatory medication within the previous 24 h that could alter pain thresholds. Additionally, they must have been treated with 0.18” Hilgers prescription metal brackets (3M Unitek, St. Paul, MN, USA) and be wearing a final stainless-steel archwire of at least 0.16” × 0.22”. Patients with cognitive disorders, craniofacial deformities, or active oral diseases that could affect periodontal health were excluded. These criteria were applied to ensure a clinically homogeneous sample appropriate for the study objectives.

Participants underwent bracket removal according to routine clinical scheduling at the time of debonding. Importantly, no prospective assignment of participants to specific interventions was performed by the investigators, as all procedures were part of routine clinical practice and determined by standard clinical scheduling. Random allocation was not applicable, as participants were managed according to routine clinical practice and were not assigned to interventions by the investigators.

All orthodontic brackets were bonded using Transbond™ XT Light Cure Adhesive Primer and Transbond™ XT Light Cure Adhesive (Solventum, formerly 3M Unitek, Monrovia, CA, USA), ensuring a standardized bonding procedure for all participants.

**Figure 1:**
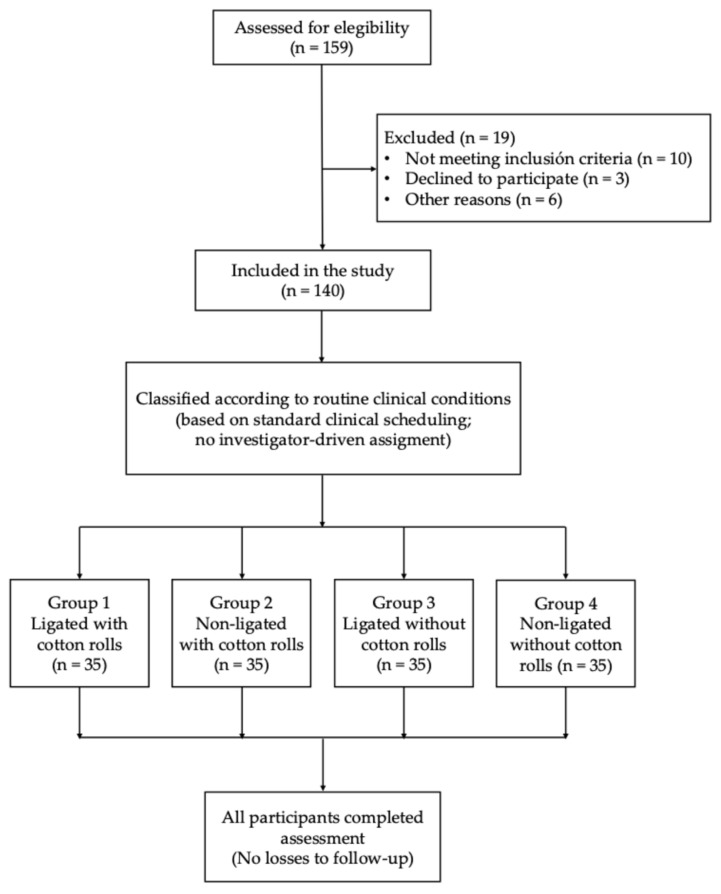
Participant selection and classification according to routine clinical practice. From 159 patients assessed for eligibility, 140 met the inclusion criteria and were classified into four groups (*n* = 35 each). No losses occurred during follow-up or analysis. Group classification was based on routine clinical practice and clinical scheduling, without investigator-driven assignment.

To minimize procedural variability, all bracket removals were performed by the same examiner. Data analysis was conducted by an independent investigator who was blinded to participant group assignment. The participant flow throughout the study is presented in Figure 1.

The bracket removal protocol was structured according to two main variables: the method of archwire ligation (with or without elastomeric ligatures) and the use or non-use of a cotton roll between the dental arches as a cushioning element during debonding. Based on routine clinical procedures, patients were classified into four groups according to the debonding conditions applied during standard care: Group 1, brackets ligated and cotton roll; Group 2, non-ligated and cotton roll; Group 3, brackets ligated and no cotton roll; and Group 4, non-ligated and no cotton roll. This distribution allowed independent and combined evaluation of the influence of both variables on pain perception during bracket removal.

Bracket removal was performed using a standardized plier to ensure procedural consistency. All participants completed a questionnaire at two time points: T0, immediately before bracket removal, and T1, immediately after the procedure.

### 2.2. Measures

At T0, the sociodemographic variables of age, sex, and educational level (no education, primary, secondary, and university education), as well as socioeconomic status (low, middle, and high), were recorded. In addition, baseline pain, anxiety, and emotional state were assessed.

Pain intensity was measured using a Visual Analog Scale (VAS) [16] ranging from 0 (no pain) to 10 (worst imaginable pain).

Anxiety was assessed using the State–Trait Anxiety Inventory (STAI) [17], a validated self-report instrument that distinguishes between state anxiety (STAI-S) and trait anxiety (STAI-T), with each subscale consisting of 20 items rated on a 4-point Likert scale. Cronbach’s alpha values [18] in the present study were 0.918 (STAI-S) and 0.873 (STAI-T).

Emotional state was measured using the Positive and Negative Affect Schedule (PANAS) [8], consisting of 20 items divided into positive affect (PA) and negative affect (NA), with each item rated on a 5-point Likert scale. Cronbach’s alpha values were 0.863 (PA) and 0.916 (NA).

All questionnaires were self-administered under the supervision of a trained psychologist, who provided standardized instructions and remained blinded to both study objectives and group allocation.

### 2.3. Statistical Analysis

Data were analyzed using IBM SPSS Statistics for Windows (version 28.0; IBM Corp., Armonk, NY, USA) [19]. Pain intensity was measured at two time points: immediately before debonding (T0) and immediately after bracket removal (T1) using a Visual Analog Scale (VAS). Descriptive statistics were computed for all study variables. Continuous variables are expressed as means and standard deviations, while categorical variables are presented as frequencies and percentages. The distribution of the data was assessed using the Kolmogorov–Smirnov test [20]. Parametric tests were applied when assumptions of normality were met, and Levene’s test was used to evaluate homogeneity of variances [21]. Given the non-randomized study design, baseline comparability among the four groups was assessed. Differences in sociodemographic variables (age, sex, socioeconomic status, and educational level), baseline pain (T0), and psychological variables were examined using one-way analysis of variance (ANOVA) [22] for continuous variables and chi-square (χ^2^) tests for categorical variables [23]. Between-group differences in pain outcomes (T0 and T1) across the four debonding protocols were initially explored using one-way ANOVA. Post hoc comparisons were performed using Scheffé’s test when statistically significant differences were identified [24]. To evaluate the independent and combined effects of cotton roll use and ligation on post-debonding pain (T1), a factorial analysis of covariance (ANCOVA) was conducted, including cotton roll use and ligation as fixed factors and baseline pain (T0) as a covariate. Additionally, a multiple linear regression model was performed to identify predictors of pain after bracket removal, including baseline pain (T0), cotton roll use, ligation status, age, and negative affect as independent variables.

The internal consistency of the psychological instruments (STAI and PANAS) was evaluated using Cronbach’s alpha coefficient. Statistical significance was set at *p* < 0.05, and effect sizes were calculated where appropriate to facilitate interpretation of the results.

### 2.4. Ethical Approval

The study protocol was reviewed and approved by the Ethics Committee of Rey Juan Carlos University (approval number: 111020231192024), in accordance with the principles of the Declaration of Helsinki.

All participants, or their legal guardians in the case of minors, received detailed information about the study and provided written informed consent prior to inclusion. Confidentiality and anonymity of participant data were strictly maintained throughout the study.

## 3. Results

### 3.1. Sample Characteristics and Baseline Comparability

The sociodemographic characteristics of the sample are presented in Table 1. The mean age of the sample was 24.74 ± 12.97 years, and the sample comprised 76 males (54.3%) and 64 females (45.7%).

No statistically significant differences were observed among the four debonding groups in terms of age, sex, socioeconomic status, or educational level (all *p* > 0.05). Similarly, no between-group differences were found for baseline psychological variables, including state anxiety (*p* = 0.249), trait anxiety (*p* = 0.473), positive affect (*p* = 0.117), and negative affect (*p* = 0.354). Descriptive statistics of the psychological variables are presented in Table 2. However, given the non-randomized design, these findings should not be interpreted as evidence of group equivalence, and residual confounding cannot be ruled out.

### 3.2. Pain During Bracket Debonding

#### 3.2.1. Between-Group Comparisons

Pain outcomes across the four debonding protocols are presented in Table 3. Baseline pain (T0) did not differ significantly among the four groups (*p* = 0.164), whereas statistically significant differences were observed in post-debonding pain (T1) and pain increase (Δ Pain). The highest pain levels were reported in Group 4 (no ligatures, no cotton roll), followed by Groups 3, 2, and 1 (ligatures with cotton roll), which showed the lowest pain scores.

#### 3.2.2. Effect of Debonding Protocol

To evaluate the independent and combined effects of debonding conditions on post-procedural pain, a factorial analysis of covariance (ANCOVA) was conducted, including cotton roll use and ligation status as fixed factors and baseline pain (T0) as a covariate.

The overall model was statistically significant (F (4,135) = 16.74, *p* < 0.001), explaining 33.1% of the variance in post-procedural pain (adjusted R^2^ = 0.312). Baseline pain (T0) was a strong predictor of post-procedural pain (F = 42.66, *p* < 0.001, η^2^ = 0.240).

A significant main effect of cotton roll use was observed (F = 9.19, *p* = 0.003, η^2^ = 0.064), with lower adjusted pain scores in patients treated with cotton rolls (M = 1.75) compared to those without (M = 2.85).

By contrast, the main effect of ligation did not reach statistical significance (F = 3.53, *p* = 0.063, η^2^ = 0.025).

The interaction between cotton roll use and ligation was not statistically significant (F = 1.29, *p* = 0.258), indicating that the effect of cotton rolls was consistent regardless of ligation status. Detailed results of the factorial ANCOVA are presented in Table 4.

### 3.3. Predictors of Pain Increase

To further examine the contribution of clinical, demographic, and psychological variables to post-debonding pain, a multiple linear regression analysis was conducted, including baseline pain (T0), cotton roll use, ligation status, age, and negative affect as predictors. The overall model was statistically significant (F (5,134) = 20.43, *p* < 0.001), explaining 43.3% of the variance in post-procedural pain (R^2^ = 0.433; adjusted R^2^ = 0.411). Baseline pain (T0) emerged as the strongest predictor of post-procedural pain (B = 0.625, β = 0.417, *p* < 0.001), and cotton roll use was independently associated with lower pain levels (B = −0.852, β = −0.166, *p* = 0.013). Age (B = 0.045, β = 0.227, *p* < 0.001) and negative affect (B = 0.089, β = 0.248, *p* < 0.001) were also significant predictors, indicating that older patients and those with higher levels of negative affect reported higher post-debonding pain. By contrast, ligation status did not reach statistical significance (B = −0.647, β = −0.126, *p* = 0.058), although a trend toward lower pain in ligated conditions was observed. The results of the multiple linear regression analysis are presented in Table 5.

These findings highlight the combined influence of mechanical and biopsychosocial factors on pain perception during orthodontic debonding.

## 4. Discussion

The influence of mechanical and psychological factors on pain perception during bracket removal was evaluated in this comparative non-randomized study. The main finding was that cotton roll use was independently associated with lower pain following bracket removal after adjusting for baseline pain, whereas ligation did not show a statistically significant independent effect. In addition, baseline pain, age, and negative affect emerged as significant predictors of post-procedural pain within a combined regression model.

While mechanical strategies have traditionally been the focus of pain reduction during orthodontic debonding, the present findings suggest that emotional variables may also play an important role in pain perception, alongside the clinical protocol [13].

The reduction in pain observed when patients were asked to bite on cotton rolls may be explained by improved force distribution and increased tooth stabilization during bracket removal. A possible explanation is that intrusive forces applied during biting are better tolerated by periodontal structures, thereby reducing discomfort during debonding. These findings are consistent with previous studies reporting that the application of intrusive forces or the use of interocclusal materials can significantly reduce pain [25,26,27]. However, not all studies have reported consistent results. For example, Kilinç et al. did not observe significant differences between debonding methods using interocclusal devices and those without, suggesting that pain perception during debonding may be influenced by multiple interacting factors beyond purely mechanical variables [28,29]. Importantly, this association remained significant after adjusting for baseline pain and other clinical and psychological variables, supporting the independent effect of cotton roll use.

Consistent with both the factorial analysis and the multivariable regression model, no statistically significant differences were observed between protocols involving ligated brackets and those without ligation. To our knowledge, limited evidence is available regarding the specific influence of ligation on pain perception during debonding. This finding may be related to the localized nature of force application during bracket removal. Even when brackets are connected via the archwire, the forces applied during removal are predominantly concentrated at the individual tooth level, potentially limiting the transmission of force to adjacent teeth. Consequently, the presence of elastomeric ligatures may have a minimal impact on the overall pain experience.

The present study also highlights the relevance of psychological factors in pain perception. Negative affect emerged as a significant predictor of post-debonding pain, which is consistent with previous evidence indicating that emotional states play a key role in modulating pain experience [30,31]. Patients with higher levels of negative affect may exhibit heightened sensitivity to nociceptive stimuli, leading to an amplified perception of discomfort. These findings are further supported by the regression analysis, in which negative affect remained an independent predictor of post-procedural pain after adjusting for clinical factors.

Age was also identified as a significant predictor of pain, with older patients reporting higher pain after bracket removal. Although evidence in orthodontics is not entirely consistent, some studies suggest that pain perception may vary across age groups due to biological and psychosocial factors, including age-related changes in pain modulation mechanisms [32,33,34]. Changes in pain modulation mechanisms, as well as differences in cognitive and emotional processing, may contribute to these variations [2]. However, conflicting findings exist, with some studies reporting higher pain levels in adolescents, indicating that the relationship between age and pain perception remains complex and warrants further investigation [35,36]. Age also remained a significant predictor in the adjusted model, reinforcing its role as an independent determinant of pain perception.

Importantly, this study contributes to the literature by integrating both mechanical and psychological determinants of pain during bracket removal within a single multivariable model, allowing estimation of their independent contributions to pain perception.

From a biopsychosocial perspective, pain perception during orthodontic procedures is influenced by biological factors [37] (e.g., mechanical variables and age), psychological variables (e.g., anxiety and negative affect), and social context. Although we integrate biological and psychological dimensions through analysis of their combined effects in the present study, other potentially relevant psychological and social variables—such as patient expectations, clinician–patient interaction, coping strategies, or the clinical environment—were not assessed. These dimensions should be considered in future research to advance toward a more comprehensive understanding of pain during the debonding process.

### 4.1. Clinical Implications

The findings of this study suggest that both procedural and patient-related factors may influence pain perception during orthodontic debonding. In particular, cotton roll use was associated with lower post-debonding pain, while negative affect emerged as an independent predictor of higher pain levels.

Although the present study did not evaluate specific psychological interventions, these findings highlight the potential importance of considering patients’ emotional state when planning and performing debonding procedures. Future controlled studies are needed to determine whether targeted communication strategies, emotional support, or other patient-centered approaches may contribute to reducing pain during orthodontic bracket removal.

### 4.2. Limitations

This study has several limitations that should be considered when interpreting the findings. First, the non-randomized design limits the ability to establish causal relationships between the studied variables and post-debonding pain. Although no statistically significant baseline differences were observed, this does not ensure equivalence among groups, and residual confounding cannot be completely excluded. Second, the wide age range of the participants may have influenced the results, as age was a significant predictor of post-debonding pain. Although age was accounted for in the statistical models, some age-related heterogeneity may remain. Third, although the analytical approach incorporated adjustment for baseline pain (T0), which strengthens the robustness of the findings, other potentially relevant clinical or contextual variables were not included in the model and may have influenced pain perception. Furthermore, the assessment of pain was limited to immediate post-debonding measurements, which do not capture potential changes in pain over time. Future studies should consider longitudinal designs to better understand the temporal dynamics of pain perception. Finally, although psychological variables such as negative affect were included, other relevant dimensions, including anxiety, coping strategies, patient expectations, and clinician–patient interaction, were not assessed. These factors may play a significant role in shaping pain perception and should be incorporated into future research.

## 5. Conclusions

Within the limitations of this non-randomized study, the use of cotton rolls during orthodontic bracket removal was associated with lower post-debonding pain after adjusting for baseline pain. By contrast, ligation status did not show a statistically significant independent effect.

Baseline pain, age, and negative affect emerged as significant predictors of post-debonding pain, highlighting the multifactorial nature of pain perception during orthodontic procedures.

These findings suggest that both mechanical and psychological factors may contribute to pain perception during bracket removal.

Further research, particularly randomized controlled studies, is needed to confirm the potential clinical value of these findings and to evaluate targeted interventions aimed at reducing pain during orthodontic debonding.

There are no patents resulting from this research.

## Figures and Tables

**Table 1:** Sociodemographic characteristics.

Variables	Total N (%) M (SD)	Debonding Protocol					
		1	2	3	4		*p*
Age (years)	24.74 ± 12.97	22.57 ± 11.78	24.34 ± 14.13	24.69 ± 12.99	27.34 ± 12.98		0.494
Sex N (%)	Female	64 (45.7%)	14 (21.9%)	17 (26.6%)	14 (21.9%)	19 (29.7%)	0.558
	Male	76 (54.3%)	21 (27.6%)	18 (23.7%)	21 (27.6%)	16 (21.1%)	
Socioeconomic status N (%)	Low	11 (7.9%)	1 (9.1%)	1 (9.1%)	3 (27.3%)	6 (54.5%)	0.136
	Middle	116 (83.5%)	33 (28.4%)	31 (26.7%)	28 (24.1%)	24 (20.7%)	
	High	12 (8.6%)	1 (8.3%)	3 (25%)	4 (33.3%)	4 (33.3%)	
Educational level N (%)	No education	3 (2.1%)	0 (0.0%)	1 (33.3%)	0 (0.0%)	2 (66.7%)	0.562
	Primary	6 (4.3%)	0 (0.0%)	2 (33.3%)	2 (33.3%)	2 (33.3%)	
	Secondary	94 (67.1%)	27 (28.7%)	24 (25.5%)	21 (22.3%)	22 (23.4%)	
	University education	37 (26.4%)	8 (21.6%)	8 (21.6%)	12 (32.4%)	9 (24.3%)	

Note. N (number of subjects), M (mean), SD (standard deviation), 1 (ligated with cotton), 2 (non-ligated with cotton), 3 (ligated without cotton), 4 (non-ligated without cotton), *p* = significance.

**Table 2:** Descriptive statistics of the variables under study.

		Protocol				
Variable	α	1 M (SD)	2 M (SD)	3 M (SD)	4 M (SD)	*p*
**STAI-S**	0.918	13.74 ± 9.77	16.86 ± 10.39	12.91 ± 11.54	17.23 ± 11.87	0.249
**STAI-T**	0.873	16.40 ± 9.32	17.57 ± 8.46	15.14 ± 9.79	18.51 ± 9.99	0.473
**PA**	0.863	35.14 ± 7.28	31.57 ± 7.12	34.86 ± 6.02	32.43 ± 8.91	0.117
**NA**	0.916	17.66 ± 6.70	16.26 ± 5.66	18.31 ± 7.05	19.26 ± 8.75	0.354

Note. α (Cronbach’s alpha for the scale), M (mean), SD (standard deviation), 1 (ligated with cotton), 2 (non-ligated with cotton), 3 (ligated without cotton), 4 (non-ligated without cotton), STAI-S (state anxiety), STAI-T (trait anxiety), PA (positive affect), NA (negative affect), *p* = significance.

**Table 3:** Comparison of the pain variables for the 4 groups under study.

	1	2	3	4	*p*
**Pain T_0_**	0.60 ± 1.3	1.14 ± 1.8	1 ± 1.7	1.5 ± 1.8	0.164
**Pain T_1_**	1.29 ± 1.88	1.94 ± 2.07	2.26 ± 2.81	3.71 ± 2.83	<0.001 **
** Δ Pain (T_1_-T_0_) **	0.68 ± 2.25	0.8 ± 2.08	1.25 ± 2.09	2.20 ± 2.29	0.018 *

Note. 1 (ligated with cotton), 2 (non-ligated with cotton), 3 (ligated without cotton), 4 (non-ligated without cotton), Pain T_0_ (baseline pain), Pain T_1_ (pain after bracket removal), *p* = significance, * = *p* < 0.05, ** = *p* < 0.001.

**Table 4:** Factorial ANCOVA examining the effects of cotton roll use and ligation on post-debonding pain (T1), adjusted for baseline pain (T0).

Effect	F	*p*	η^2^
**Baseline pain (T0)**	42.66	<0.001	0.240
**Cotton roll use**	9.19	0.003	0.064
**Ligation**	3.53	0.063	0.025
**Cotton × Ligation**	1.29	0.258	0.009

*Note.* ANCOVA (Analysis of Covariance); T0 = baseline pain (included as a covariate); F = Snedecor’s F-statistic; *p* = statistical significance level (*p* < 0.05 is considered statistically significant); η^2^ = partial eta squared (measures the effect size, where 0.01 indicates a small effect, 0.06 a medium effect, and 0.14 a large effect).

**Table 5:** Multiple linear regression analysis predicting pain (T1).

Predictor	B	SE	β	t	*p*
**Constant**	−0.329	0.641	–	−0.513	0.609
**Pain T0**	0.625	0.101	0.417	6.166	<0.001
**Cotton roll use**	−0.852	0.339	−0.166	−2.510	0.013
**Ligation**	−0.647	0.339	−0.126	−1.909	0.058
**Age**	0.045	0.013	0.227	3.462	<0.001
**Negative affect**	0.089	0.024	0.248	3.708	<0.001

*Note*: Dependent variable: pain after bracket removal (T1). B = unstandardized coefficient; β. Model summary: R = 0.658, R^2^ = 0.433, Adjusted R^2^ = 0.411, F (5,134) = 20.43, *p* < 0.001.

## Data Availability

The datasets presented in this article are not readily available because of ethical reasons.

## Abbreviations

The following abbreviations are used in this manuscript:

T_0_	baseline pain
T_1_	pain after bracket removal
Δ Pain (T_1_-T_0_)	pain increase
1	Group 1. Ligated with cotton
2	Group 2. Non-ligated with cotton
3	Group 3. Ligated without cotton
4	Group 4. Non-ligated without cotton
NA	Negative affect
PA	Positive affect
